# Burden of Disease Attributed to Prenatal Methylmercury Exposure in the Yanomami Indigenous Land

**DOI:** 10.3390/toxics13050339

**Published:** 2025-04-25

**Authors:** Ana Claudia Santiago de Vasconcellos, Raiane Fontes de Oliveira, Marcos Wesley Oliveira, Paulo Cesar Basta

**Affiliations:** 1 Laboratory of Professional Education in Health Surveillance, Joaquim Venâncio Polytechnic Health School, Oswaldo Cruz Foundation, Rua Leopoldo Bulhões 1480, Rio de Janeiro 21041-210, RJ, Brazil; 2 Rio Negro Program, Socio-Environmental Institute (ISA)—Pç. Dom José Gaspar, 134 (12th Floor), São Paulo 01047-912, SP, Brazil; marcos@socioambiental.org; 3 Department of Endemic Diseases Samuel Pessoa, National School of Public Health, Oswaldo Cruz Foundation, Rua Leopoldo Bulhões 1480, Rio de Janeiro 21041-21, RJ, Brazil; paulobasta@gmail.com

**Keywords:** mild mental retardation, illegal gold mining, disability-adjusted life years

## Abstract

The Yanomami Indigenous Land (YIL) is heavily impacted by illegal gold mining, leading to significant contamination by methylmercury, a neurotoxin that poses severe risks to human health. The fetal brain is particularly susceptible to the neurotoxic effects of methylmercury, which can result in mild mental retardation (MMR). The goal of this study was to estimate the burden of disease (BoD) associated with methylmercury exposure in the YIL and its economic implications. The BoD calculations followed World Health Organization (WHO) methodologies. To estimate the local BoD, hair samples were collected from women of childbearing age in the Waikás, Mucajaí, Paapiu, and Maturacá regions. For broader estimates, data from the scientific literature were used. The average hair methylmercury concentrations in these investigated regions were 6.21 µg/g, 3.86 µg/g, 3.53 µg/g, and 2.96 µg/g, respectively. The MMR incidence rate (IR) in children ranged from 2.08 to 4.47 per 1000 in these regions. The Disability-Adjusted Life Years (DALYs) per 1000 births varied from 24.8 to 53.4. In the Worst-Case Scenario, MMR-IR reached 9 per 1000, with DALYs per 1000 births rising to 109.6. The estimated economic impact of methylmercury exposure ranged from USD 716,750 to USD 3,153,700. This study is the first to quantify the MMR incidence due to mercury in the YIL, highlighting the severe threat posed by gold mining to the health and survival of the Yanomami people.

## 1. Introduction

The gold mining in the Yanomami Indigenous Land (YIL) aroused the concern of the national and international scientific community in the 1980s when the increase in the price of gold on the international market led to a huge rush in search of gold throughout the Amazon region [1,2]. Years later, in 1990, Selva Livre Operation, organized by the Federal Government, led to the expulsion of thousands of miners from the region [1]. However, from the 2000s onwards, far-right governments allowed and encouraged the expansion of illegal gold mining in the Yanomami territory and other Indigenous lands in the northern region of the country, making the scenario of environmental and social destruction caused by mining catastrophic [3].

One of the most terrifying repercussions of gold mining is the environmental contamination caused by mercury. Recent studies indicate that thousands of mercury tons have already been deposited into the Amazon ecosystem as a result of gold extraction activities [1,4]. Once released into aquatic systems, elemental mercury (Hg^0^) undergoes transformation into methylmercury through the action of microorganisms. This organic mercury form bioaccumulates in all organisms within the aquatic biota and contaminates humans through feeding [5,6].

Grandjean and Landrigan [7] carried out a study that provided evidence of the high susceptibility of the central nervous system to methylmercury, particularly emphasizing a fetus’s vulnerability during pregnancy. Consequently, the ingestion of fish and other aquatic organisms containing elevated levels of methylmercury presents a significant risk to pregnant women, potentially compromising the neurological development of the fetus [8].

Human brain exposure to methylmercury can result in cognitive impairments, leading to diminished intelligence and mental retardation. As defined by the American Association of Mental Retardation, this diagnosis is characterized by low IQ scores and impairments in adaptive functioning [8,9]. Mental retardation manifests across various degrees of severity, with approximately 85% of cases classified as mild mental retardation (MMR), often of idiopathic origin. MMR stands as one of the prevalent psychiatric disorders among children and adolescents, with prevalence rates estimated to range from 1 to 3% within the population [9].

Assessing the human health consequences of mercury exposure includes estimating the burden of disease (BoD) linked to this risk factor. The BoD acts as a metric used to measure the gap between the ideal scenario, where individuals enjoy optimal health until old age, and the current reality, where health decline or mortality occurs due to various causes, such as traffic accidents, infectious diseases, and exposure to environmental pollutants [10]. This estimation holds fundamental significance in informing decision-making processes, prioritizing efforts, and allocating financial resources for scientific research investments or intervention initiatives.

Presently, the metric employed for quantifying the burden of disease (BoD) is the Disability-Adjusted Life Year (DALY) metric, which serves as an indicator of the overall health status within a population [10,11].

In addition to methylmercury exposure, Yanomami communities face numerous other risk factors, including inadequate sanitation, limited access to safe drinking water, chronic undernourishment, and high incidences of infectious diseases [12]. Hence, there is a pressing need to conduct studies aimed at assessing the health repercussions specifically associated with methylmercury exposure. Such investigations can serve as a foundation for prioritization efforts and the development of public health policies geared towards the prevention and control of mercury exposure.

The principal objective of this study was determining the incidence rate of mild mental retardation (MMR) among Yanomami children who were exposed to methylmercury during the prenatal period. Additionally, this study aimed to quantify the burden of disease (BoD) attributed to this risk factor utilizing the Disability-Adjusted Life Year (DALY) metric and the monetized impact associated with the healthy years of life lost, following methodologies proposed by the World Health Organization (WHO).

## 2. Materials and Methods

### 2.1. Study Area and Population

Yanomami Indigenous groups are known for their hunter–gatherer lifestyle and traditional slash-and-burn (*coivara*) farming practices, residing within the Amazon tropical forest. Their territory spans the Massif of the Guianas, crossing the borders of Brazil and Venezuela, covering a total area of 192,000 km^2^. The estimated population of Indigenous people living within the Yanomami Indigenous Land in Brazil today is around 31,000 individuals. These Indigenous communities are representatives of six Yanomami subgroups, as well as two isolated groups, in addition to the Ye’kwana people. They are distributed across approximately 384 villages located between the states of Roraima and Amazonas [13].

**Figure 1:**
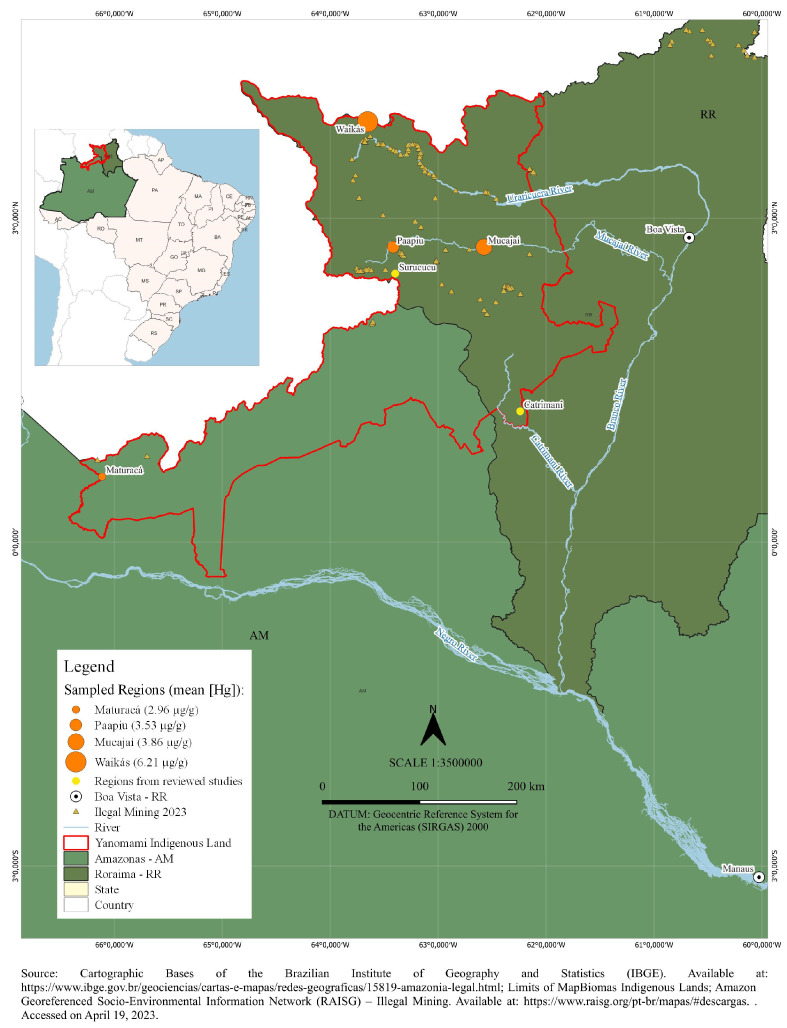
Yanomami Indigenous Land.

This research was conducted within the Brazilian territory, specifically in four areas of the Yanomami Indigenous Reserve named Waikás, Mucajaí, Paapiu, and Maturacá. These areas are located in the northwest of the Amazon region (see Figure 1), representing approximately 30 villages.

### 2.2. Sample Collection and Mercury Analysis

Following the recommendations outlined by the World Health Organization [14] and supported by additional research [15,16], hair samples are recognized as the most reliable biomarker for evaluating human exposure to methylmercury (MeHg). This is primarily due to the fact that the vast majority of mercury found in hair is present as methylmercury. In the context of this study, where fish consumption is the predominant source of MeHg exposure and where mercury in fish muscle tissue is almost exclusively in the methylmercury form, hair sampling was deemed the most appropriate method for exposure assessment [5,6]. Therefore, it was assumed that the mercury levels detected in the hair samples represented exposure solely to methylmercury.

Hair samples were collected from women of reproductive age (15–49 years) living in Indigenous communities across four regions: Waikás, Paapiu, Maturacá, and Mucajaí (see Figure 1). Sampling in the Waikás and Paapiu areas was conducted in December 2014, followed by collection in Maturacá in February 2019, and in Mucajaí in October 2022.

To be included in this cross-sectional study, the participants had to meet three criteria: (i) continuous residence in the community for at least 12 months; (ii) voluntary participation confirmed through signed informed consent; and (iii) agreement to provide hair samples. The study protocol and procedures received ethical approval from the Ethics Committee of the National School of Public Health, Oswaldo Cruz Foundation.

Hair was taken from the occipital region using stainless steel dissection scissors and stored in individually labeled paper envelopes for identification. The samples were then sent to specialized laboratories for analysis of total mercury (THg) concentrations.

The samples from the Waikás and Paapiu communities were processed in the Chemistry Department laboratory at Pontifícia Universidade Católica in Rio de Janeiro. Approximately 0.1 g of hair was weighed, treated with 1 mL of purified nitric acid (HNO_3_) at room temperature for 12 h, and then heated at 80 °C for one hour. Hydrogen peroxide (0.4 mL) was subsequently added, followed by another heating phase at the same temperature for 30 min. Rigorous quality assurance measures were in place, including daily blank controls and calibration curves. Certified Reference Material (CRM-13) for human hair was used to assess accuracy, with recovery rates exceeding 90%. All analyses were conducted using Inductively Coupled Plasma Mass Spectrometry (ICP-MS 7500 CX, Agilent Technologies, Hanover, Germany) [17].

The hair samples from the Maturacá and Mucajaí communities were analyzed at the Toxicology Laboratory within the Environmental Section of the Instituto Evandro Chagas (IEC), located in Ananindeua, Pará, Brazil. Prior to analysis, the hair samples were washed using a 100-fold diluted neutral detergent (Extran, Merck KGaA, Darmstadt, Germany), dried, and then cut into a fine, powder-like state to ensure homogeneity. For analysis, between 10 and 20 mg of the hair was used. The procedure included chemical digestion and reduction with a 10% SnCl_2_ solution, followed by measurement using cold vapor atomic absorption spectrometry (CVAAS) at 253.7 nm (Hg-201 Semi-automated Mercury Analyzer, Sanso Seisakusho Co., Ltd., Tokyo, Japan). Quality control measures involved (i) the use of method blanks; (ii) calibration with a 6-point curve (0.4–4 ng/g); (iii) analysis of IAEA-86 Human Hair Certified Reference Material with an average recovery of 101% (range: 83.4–106.6%, *n* = 8); and (iv) monitoring of the relative standard deviation (RSD), which was 8.32%. Random sample replicates (*n* = 10) yielded an RSD of 2.49%. The limits of detection and quantification were 0.0083 ng/mg and 0.027 ng/mg, respectively [18].

### 2.3. Data Collection to Estimate Exposure in Yanomami Indigenous Land

Information regarding methylmercury exposure in various Yanomami communities was gathered through a review of the scientific literature using databases such as PubMed, Web of Science, and Google Scholar. The search was conducted using the keywords “mercury” OR “methylmercury” in combination with “Yanomami” within the topic field. This review aimed to locate peer-reviewed studies that reported mercury concentrations in hair or blood samples of women residing in different regions of the Yanomami Indigenous Territory. We only considered the studies that fulfilled the following inclusion criteria: (i) published in Portuguese, Spanish, or English; (ii) original research articles appearing in peer-reviewed scientific journals; and (iii) freely accessible for download.

### 2.4. Methylmercury-Attributed BoD

Methylmercury is known to primarily affect the central nervous system, with the fetal stage being especially susceptible to harm from xenobiotic substances. One of the key health outcomes associated with prenatal exposure to methylmercury is cognitive impairment in children [19,20]. This impact is typically assessed by measuring the intelligence quotient (IQ), using standardized tests to identify potential deficits. Although a reduction in IQ is not classified as a disease, it contributes to mild mental retardation (MMR), which is used as a measurable outcome of prenatal methylmercury exposure [19,20].

For the purpose of estimating the burden of disease (BoD) related to prenatal methylmercury exposure, MMR is considered the primary outcome. This approach relies on a specific dose–response relationship documented in the literature, which suggests that for each 1.0 µg/g of mercury detected in maternal hair, there is an average IQ loss of 0.18 points in the offspring. This model assumes a linear, no-threshold association between the level of mercury in maternal hair and IQ loss in children [19].

It is also important to consider that IQ scores in the general population follow a normal distribution, with a mean of 100 and a standard deviation of 15 points [9]. MMR is typically characterized by IQ scores ranging from 50 to 70 [9]. To estimate how many children fall into this range due to maternal exposure to methylmercury, calculations are based on the mean and standard deviation of mercury concentrations found in the hair samples collected from the study population [10].

### 2.5. Method to Estimate the BoD Attributed to Prenatal Methylmercury Exposure

To estimate the burden of disease (BoD) resulting from prenatal exposure to methylmercury, the health impact was measured using Disability-Adjusted Life Years (DALYs). DALYs combine two components: years of life lost (YLL) due to premature mortality and Years Lived with Disability (YLD). In this analysis, the following parameters were used for DALY estimation: full age weighting (value of 1 or 100%), a 3% discount rate, the incidence of mild mental retardation (MMR), a disease weight (DW) of 0.361, and an illness duration aligned with standard life expectancy—80 years for men and 82.5 years for women [10].

Age weighting in DALY calculations reflects societal judgments about the relative value of life years at different ages. Generally, years in childhood or old age receive lower weights due to lower assumed productivity. However, for MMR due to prenatal methylmercury exposure, a full weight (1.0) was applied. This reflects the fact that the condition affects the individual’s entire life, regardless of age group [10].

The approach is based on the premise that methylmercury-induced damage to the developing brain is permanent, leading to a lifelong reduction in IQ [10]. Since a specific disease weight for MMR caused by methylmercury does not exist, the disease weight developed for lead-induced MMR (0.361) was used as a proxy in this context [10].

Additionally, a 3% annual discount rate was applied when projecting years of life lost into the future. This practice is common in public health assessments, as it gives relatively less weight to future health losses compared to immediate ones [10]. The incidence of MMR per 1000 live births was calculated based on the mean and standard deviation of hair mercury levels measured in women of reproductive age. The total DALYs, representing healthy life years lost, were then estimated by multiplying this incidence by the number of births within the study population. Since MMR does not result in premature death, the DALY here corresponds exclusively to YLD.

DALY computations were conducted using custom Microsoft Excel tools developed by the World Health Organization [10] and shared by A. Prüss-Üstün (personal communication). One spreadsheet estimated the percentage of infants affected by a 2.0 IQ point loss and the incidence of MMR per 1000 births, while the second calculated total DALYs based on these estimates and the number of births in the population.

Within the first spreadsheet, the Excel function NORMDIST was used to estimate the proportion of the population with hair mercury concentrations exceeding 10 µg/g. Based on the dose–response relationship proposed by Axelrad et al. [19], this level corresponds to a loss of 1.98 IQ points. The NORMDIST (x, µ, σ, cumulative) function returns the cumulative probability that a normally distributed variable with mean µ and standard deviation σ will be less than or equal to x. Therefore, 1–NORMDIST (x, µ, σ, 1) was used to calculate the fraction of the population exceeding a certain mercury threshold. This method assumes that mercury concentrations in hair, like human IQ, are normally distributed in the exposed population.

### 2.6. Local Estimates of BoD Attributed to Prenatal Methylmercury Exposure

To assess the burden of disease (BoD) at the local level, primary data on methylmercury concentrations in hair samples from women of reproductive age residing in the communities of Waikás, Paapiu, Maturacá, and Mucajaí were utilized. Specifically, the analysis was based on the mean values and standard deviations of mercury levels detected in these samples. For the calculation of DALYs, an estimate of 1000 live births was used, representing the average annual number of births within the Yanomami Indigenous Territory between 2019 and 2023, according to data provided by the Yanomami Indigenous Special Health District (DSEI-Yanomami).

### 2.7. BoD Attributed to Prenatal Methylmercury Exposure in the YIL

The burden of disease (BoD) for the entire Yanomami Indigenous Land (YIL) was estimated using the average values and standard deviations of methylmercury concentrations found in hair or blood samples, as reported across all studies identified during the literature review. For each study, we calculated the following metrics: (i) the percentage of infants estimated to experience a 2.0-point IQ reduction; (ii) the incidence rate of mild mental retardation (MMR); and (iii) DALYs per 1000 live births.

Based on the compiled data, three distinct exposure scenarios were developed to reflect the potential range of methylmercury exposure at the regional level. Additionally, the DALY calculations incorporated demographic data provided by the DSEI-Yanomami (Yanomami Indigenous Special Health District).

### 2.8. Construction of Alternative Scenarios and Counterfactual Analysis

Following the methodology proposed by Murray and Lopez [11] for estimating the burden of disease (BoD) in general populations, it is essential to develop alternative scenarios that reflect different levels of exposure to the risk factor under investigation, ranging from minimal to elevated or even absent exposure. In line with this approach, three exposure scenarios for methylmercury were established: Best-Case, Intermediate, and Worst-Case Scenarios. These scenarios were derived from the mercury concentrations in the hair samples reported across the studies identified during the literature review.

### 2.9. Monetized Impact of IQ Loss

The financial quantification of DALYs resulting from mercury exposure has been the focus of several studies [21,22,23], which have proposed that one DALY represents the annualized value of a statistical life. As a result, the monetary value of DALYs can reach upwards of USD 200,000 per DALY [24]. Our methodology follows the World Health Organization’s guidance [25], which states that the value of one year of healthy life lost, as measured by DALYs, is equivalent to three times the Gross Domestic Product (GDP) per capita. In Brazil, this figure amounted to USD 28,670 in 2023 [26].

## 3. Results

### 3.1. Hair Mercury Levels in Yanomami Women (Primary Data)

A total of 317 women of childbearing age from four Yanomami regions took part in this study. The concentrations of methylmercury detected in hair samples, according to the minimum and maximum levels, percentiles (25th, 50th, 75th, and 95th), and their distribution across the four Indigenous regions studied are presented in Table 1. The widest ranges of methylmercury levels were found among women from Waikás (ranging from 2.00 to 19.75 µg/g) and Maturacá (ranging from 0.53 to 10.26 µg/g).

### 3.2. Local Burden of MMR

Women living in the Waikás and Mucajaí regions had the highest average methylmercury concentrations in their hair, with values of 6.21 and 3.86 µg/g, respectively. In contrast, the Maturacá region exhibited the lowest concentration at 2.96 µg/g. Using the mean and standard deviation of these methylmercury levels, the incidence of mild mental retardation (MMR) was estimated (see Table 2). In the Waikás region, approximately 15.41% of the newborns were predicted to experience a reduction of 2.0 IQ points, resulting in an incidence rate of 4.47 per 1000 live births. As a result, the DALYs per 1000 inhabitants in this region were calculated to be 53.4 (refer to Table 2 and Table 3).

### 3.3. Burden of MMR in the YIL

Given the wide range of methylmercury concentrations found in the hair samples from Yanomami communities (Table 3), we conducted a counterfactual analysis to estimate the burden of disease (BoD) within the Yanomami Indigenous Land (YIL). This analysis considered three distinct exposure scenarios. The “Best Scenario” assumed low levels of methylmercury exposure (<3 µg/g), with a lower risk for delays in child neurodevelopment [4,5,6]. In the “Intermediate Scenario”, exposure levels ranged from 3 µg/g to 10 µg/g, which is considered the threshold at which neurological damage may begin to affect unborn children [4,5,6]. The “Worst-Case Scenario” involved average methylmercury levels exceeding 10 µg/g, where the risk for neurological damage is high (Table 4).

The “Best Scenario” was based on data from women in the Maturacá region, where the average methylmercury concentration was below 3.0 µg/g (primary data). In this scenario, the incidence rate of mild mental retardation (MMR) was 2.08 per 1000 infants, resulting in 24.8 DALYs per 1000 inhabitants, with a total of 25 DALYs. No decrease in IQ was predicted for this population.

The “Intermediate Scenario” used data from Koremutheri Village in the Surucucu region, as reported by Castro et al. [2]. Here, the mean methylmercury concentration was 5.01 µg/g. The MMR incidence rate was calculated at 3.41 per 1000, which resulted in 40.7 DALYs per 1000 and a total of 41 DALYs.

The “Worst-Case Scenario” was based on the study by Sing et al. [27], which examined the Maamapiitheri community along the Catrimani River Basin. In this case, the average mercury concentration in hair was 12.18 µg/g. It was estimated that nearly 80% of the children born would experience a reduction of 2.0 IQ points. The MMR incidence rate in this scenario was 9.18 per 1000 live births, resulting in 109.6 DALYs per 1000.

### 3.4. Monetary Impacts of DALYs

The scenarios constructed for counterfactual analysis revealed that the monetary impact associated with MMR varied from USD 716,750 to USD 3,153,700 (Table 4).

## 4. Discussion

In this study, the BoD methodology was used for the first time to estimate the incidence of MMR attributed to chronic exposure to methylmercury. This represents a significant advancement in understanding the health impacts of environmental contaminants on Indigenous populations in the Brazilian Amazon. This approach allowed us to quantify the burden of mental retardation associated with methylmercury exposure, providing valuable insights into the scale of the problem and its financial implications for affected individuals and communities.

Despite the widespread presence of mercury throughout the Amazon region and the increasing activity of gold mining in the YIL in recent years, significant variations in methylmercury exposure levels were observed across the different regions of the Yanomami land studied in this work.

One possible explanation for the observed discrepancies in mercury exposure across the different communities is the variation in dietary habits. While Yanomami communities typically consume similar staple foods—such as fish as the primary source of protein, cassava and various roots for energy, and tropical fruits—these food items vary seasonally and geographically [5]. Despite these variations, the ongoing release of mercury into the environment due to gold mining activities continues to pose significant risks to both human health and the surrounding ecosystem.

In addition, the proximity of communities to gold mining areas plays a pivotal role in determining the level of human exposure [6,17,18]. The methylmercury concentrations in the hair samples from the Indigenous communities studied (primary data) exhibited considerable variation, ranging from 2.96 to 12.18 µg/g. As a result, the DALY rates per 1000 individuals also showed significant variation, ranging from 24.8 to 109.6, depending on the level of mercury exposure considered.

Using the DALY rates derived from primary data, we estimated the rates of DALYs per 1000 infants, which ranged from 25 to 53 (Local BoD), while the regional BoD rates varied from 24.8 to 109.6 per 1000 infants, depending on the exposure scenario outlined in the counterfactual analysis. To better understand the impact of methylmercury as a health risk factor for the Yanomami population, it is essential to compare these results with other BoD estimates related to methylmercury exposure.

To date, our research team has identified only one other study employing a similar approach within an Amazonian population [8]. This study estimated the BoD linked to methylmercury contamination in women living along the Madeira River in Rondônia state. The average methylmercury concentration in their hair was 6.49 µg/g, leading to an incidence rate of mild mental retardation of 5.96 per 1000 births and a DALY rate of 71.2 per 1000 (total DALY of 576).

Comparing the outcomes of the study involving women from the Madeira River region with those of Yanomami women, it is evident that cognitive impairment in the Madeira region appears to be more pronounced than in all Yanomami communities analyzed in our study (including primary and secondary data), except for the Maamapiitheri community. Indeed, the longstanding gold mining activity in the Madeira River basin, dating back to the 1980s, is likely to have significant and far-reaching health impacts on the local population due to chronic exposure to mercury, when compared to the Yanomami situation.

In contrast, Lackner et al. [28] examined women residing in Germany, where mercury emission sources are diffuse and do not pose a pollution risk comparable to the gold mines in the Amazon. In their study, the average mercury concentration in hair was 0.865 µg/g (ranging from 0.00622 to 8.03 µg/g). It was estimated that the MMR incidence rate per 1000 births was 0.752, with a DALY rate of 20 per 1000.

The recent study conducted by Jacques et al. [29] corroborates the findings of our work, as it reveals that among 58 Yanomami-Ninam children assessed by two neurological tests, the average Total Intelligence Quotient (TIQ) was 68.6, with a range from 42 to 92 points (median: 69.5; standard deviation: 10.5). It is noteworthy that in ideal conditions, the average TIQ is around 100 points. Additionally, the authors demonstrated that low IQ test scores were associated with a fivefold increase in the risk of having high levels of mercury in hair samples.

There is no denying the severity of the methylmercury environmental issue within the Yanomami territory. However, utilizing local monitoring data for extrapolative calculations across the entire Yanomami territory may not be appropriate due to significant variations in mercury levels between Yanomami communities. For instance, the levels observed in the women from the Maturacá Region (2.96 µg/g) do not indicate a high risk, whereas in some communities along the banks of the Catrimani River, the levels exceed 6.0 µg/g, posing a significant health risk. Nonetheless, further studies within Yanomami communities remain crucial, particularly for identifying vulnerable population groups.

The calculation of the monetary impact resulting from the loss of IQ in Yanomami Indigenous people unveiled losses spanning from USD 716,750 to USD 3,153,700. The decline in intelligence not only hampers individual academic progression but also contributes to reduced life expectancy, fosters antisocial behavior and the abuse of alcohol and illegal substances, and heightens the propensity for criminal activity [22].

The estimates of the BoD attributed to various sources of pollution serve as crucial tools for crafting public health policies and managing the available resources for health activities and services. Such estimates are essential for raising awareness about the risks associated with environmental pollution and can serve as the foundation for policy interventions. Despite the advantages of using this innovative methodology, it is important to acknowledge some limitations. Specifically, concerning the assessment of maternal MeHg exposure impacts, this methodology primarily focuses on neurodevelopmental damage to the fetus while neglecting other potential health effects, such as cardiovascular damage. Moreover, it predominantly measures the loss in IQ without fully accounting for broader neurological impairments that may affect other brain functions. Another limitation is its failure to consider potential reductions in IQ due to other maternal risk factors, such as alcoholism, thyroid disorders, malnutrition, and exposure to lead. Additionally, DALYs (Disability-Adjusted Life Years) also have limitations as population health indicators. This methodology also does not take into account that the consumption of selenium-rich foods may reduce mercury toxicity, nor that the regular intake of fish rich in polyunsaturated fatty acids may exert a protective effect on the cells of the central nervous system. It oversimplifies complex realities and depends on subjective social values, like disability weights and age weights. Decision-makers must recognize these uncertainties, along with the challenges posed by multiple causes, co-morbidities, and inconsistencies in cause–effect relationships.

Despite the potentially severe health consequences of mercury contamination for the Yanomami people and other Indigenous groups in Brazil, effective territorial protection strategies remain absent. Fundamental measures—such as the permanent removal of intruders and exploiters of natural resources (e.g., gold miners, loggers), the legal prosecution of those truly responsible for illegal activities on Indigenous lands, and continuous monitoring to prevent the return of these actors—have yet to be fully implemented. In the field of public health, the implementation of structured health surveillance actions is essential. These should include the training of healthcare professionals to enable them to recognize and manage cases of mercury exposure, the continuous monitoring of mercury levels in populations living in areas influenced by gold mining, and the development of preventive strategies focused on nutritional guidance, particularly during the prenatal period and early childhood. Recommendations should emphasize the consumption of selenium-rich foods and provide guidance on limiting the intake of carnivorous fish. This type of nutritional guidance holds significant value, as the consumption of selenium-rich foods, such as Brazil nuts (i.e., Castanha do Pará), for example, may play a protective role against the toxic effects of mercury on the human body [30,31,32]. On the other hand, the consumption of carnivorous fish, such as piranha, tucunaré, and pirarucu, increases the risk of illness due to the high mercury levels commonly detected in these species.

In this context, the importance of studies such as the present one is underscored by rigorously analyzing the direct impacts of mercury contamination and providing clear and accessible data. Such studies support policymakers and public administrators in the development of effective measures aimed at addressing the health needs of Indigenous populations.

## 5. Conclusions

This study marks the first effort to generate consistent data regarding the MMR incidence rates and intelligence loss resulting from prenatal exposure to methylmercury among the Yanomami Indigenous people. It has enabled the estimation not only of the compromised quality of life among the Yanomami residing in regions influenced by mining activities but also the quantification of years of healthy life lost and the monetary costs attributed to productivity decline. Given that mercury released by mining persists in the environment for over a century and that intelligence loss is irreversible and linked to reduced life expectancy, the encroachment of gold mining in the Yanomami territory undoubtedly stands as the greatest threat to the survival and well-being of the Yanomami people today.

Finally, our findings add valuable insights to the understanding of the complex interactions between environmental factors, neurodevelopment, and health outcomes among Indigenous communities. Our results underscore the importance of interdisciplinary approaches that integrate not only environmental science, public health, and clinical–neurologic assessment but also health economic concepts to address the multifaceted challenges faced by vulnerable populations exposed to environmental contaminants.

## Figures and Tables

**Table 1:** Distribution of methylmercury (μg/g) in the hair of women from Yanomami Indigenous Land regions.

Region	*n*	Min	P25	P50	P75	P95	Max
Waikás	29	2.00	4.12	4.85	6.70	12.73	19.75
Paapiu	66	0.56	2.33	3.51	4.35	6.60	7.40
Maturacá	131	0.53	1.83	2.65	3.75	5.68	10.26
Mucajaí	91	1.14	2.69	3.70	5.26	6.63	7.50

Min = minimum; Max = maximum; P = percentile.

**Table 2:** Mild mental retardation incidence rate in children from Yanomami Indigenous Land regions.

Region	*n*	Mean	SD	IR (Per 1000 Infants)
Waikás	29	6.21	3.72	4.47
Paapiu	66	3.53	1.56	2.45
Mucajaí	91	3.86	1.57	2.67
Maturacá	131	2.96	1.60	2.08

SD = standard deviation; IR = incidence rate.

**Table 3:** Burden of disease in different locations of the Yanomami Indigenous Land.

Reference	Region/Village		Mean (SD)	% Losing 2.0 IQ pts	MMR-IR/1000 Infants	DALYs/1000	DALYs
Castro et al.	Surucucu Region	Koremutheri	5.01 (1.04)	0.0	3.41	40.7	41
		Maisibiu	5.03 (1.67)	0.15	3.43	40.9	41
		Kanau	3.40 (0.73)	0.0	2.40	28.7	29
		Xahori	3.99 (1.46)	0.0	2.76	32.9	33
Sing et al.	Catrimani River Basin	Haximapiiporatheri	6.13 (2.43)	5.56	4.25	50,7	51
		Wapokohipiitheri	4.24 (1.78)	0.06	2.92	34.9	35
		Ukuxipiitheri	8.08 (3.45)	28.89	5.83	69,6	70
		Maamapiitheri	12.18 (2.53)	80.56	9.18	109.6	110
		Rohahipiitheri	6.58 (1.75)	2.53	4.52	54.0	54
Present Study	Waikás Region		6.21 (3.72)	15.41	4.47	53.4	53
	Paapiu Region		3.53 (1.56)	0.0	2.45	29.2	29
	Maturacá Region		2.96 (1.60)	0.0	2.08	24.8	25
	Mucajaí Region		3.86 (1.57)	0.0	2.67	31.9	32

SD = Standard Deviation; IQ = Intelligence Quotient; Pts = Points;MMR-IR = Mild Mental Retardation Incidence Rate; DALY = Disability-Adjusted Life Year.

**Table 4:** Methylmercury exposure scenarios for contrafactual analysis.

	Best Scenario (Maturacá Region)	Intermediate Scenario (Koremutheri Village/Surucucu Region)	Worst Scenario (Maamapiitheri Village/Catrimani River Region)
Mean *	2.96	5.01	12.18
SD	1.60	1.04	2.53
MMR-IR/1000 infants	2.08	3.41	9.18
DALY/1000	24.8	40.7	109.6
DALY Total	25	41	110
Number of Birth Loss 2.0 pts IQ (%)	00	0.0	806 (80.56)
Monetary Impact (USD)	716,750	1,175,470	3,153,700

* Mean of methylmercury levels in hair samples; SD = Standard Deviation; IQ = Intelligence Quotient; Pts = Points; MMR-IR = Mild Mental Retardation Incidence Rate; DALY = Disability-Adjusted Life Year.

## Data Availability

The data cannot be disclosed due to confidentiality reasons.
